# Comparison of *suansun* fermentation methods based on SBSE-GC-MS combined with SVM machine learning

**DOI:** 10.3389/fmicb.2025.1598252

**Published:** 2025-07-15

**Authors:** Jianwen Wu, Yizhao Li, Mi Qiu, Jihua Guan

**Affiliations:** ^1^Guangxi Laboratory of Forestry, Guangxi Forestry Research Institute, Nanning, China; ^2^Faculty of Agricultural Engineering, Guangxi Vocational and Technical College, Nanning, China

**Keywords:** SBSE, *suansun*, SVM, 16sRNA, microbial community

## Abstract

**Introduction:**

This study aimed to analyze the flavor profile and microbial community structure of 54 *Suansun* samples, fermented using three different methods: direct fermentation, natural water-sealed fermentation, and natural fermentation. The combination of SBSE-GC-MS, electronic nose, 16S rRNA, and SVM machine learning was used for comprehensive discrimination.

**Methods:**

The flavor components and microbial community structure were analyzed using SBSE-GC-MS, electronic nose, and 16S rRNA sequencing. SVM machine learning was employed to classify the samples based on their characteristics.

**Results:**

A total of 114 common aroma components were identified, including esters, alcohols, hydrocarbons, ketones, acids, aldehydes, heterocyclic compounds, phenols, halogenated hydrocarbons, amides, and others. Using a *p* < 0.05 and VIP > 1 threshold, 27 key characteristic flavor compounds were identified, with the highest concentration found in the natural water-sealed fermentation method. The SVM model achieved a 100% discrimination rate. Dominant bacterial genera identified across the methods were *Lactiplantibacillus*, *Lactococcus*, *Weissella*, and *Limosilactobacillus*, with a 95.65% match between dominant genera and key flavor compounds in natural water-sealed fermentation.

**Discussion:**

The study highlights that natural water-sealed fermentation is the most effective method for enhancing flavor profiles, and that *Weissella* plays a significant role in the production of key flavor compounds, particularly *p*-cresol, which increased over 600 times in natural water-sealed fermentation. Direct fermentation significantly shortens the fermentation cycle, while natural water-sealed fermentation offers the best results in terms of flavor development.

## 1 Introduction

Sour bamboo shoots (*suansun*) are a fermented vegetable product with a unique flavor. *Suansun* are used in many local dishes, such as Liuzhou Luosifen, Laoyou powder, Guilin rice noodles, and other traditional dishes, including northern Guangdong roast duck. *Suansun* are also popular among ethnic minorities in India, Southeast Asia, regions south of the Himalayas, Nepal, Bhutan, Thailand, and Taiwan in China (Choudhury et al., 2012). In 2023, the sales revenue of the whole industrial chain of Luosifen in Liuzhou, China reached 66.99 billion yuan, up 11.5% year on year. In particular, during the COVID-19 pandemic, Luosifen became even more popular as a home food. The daily average production of Luosifen alone has reached 5.06 million bags. Bagged products produced by the industry have been sold all over the world, leading to a huge demand for *suansun*. Fresh bamboo shoots are prone to lignification after harvesting, which leads to deterioration of edibility and processing quality. Lignification is characterized by the accumulation of cellulose, hemicellulose, and lignin, resulting in increased firmness, decreased water content, and reduced porosity of the tissue. The process is associated with transcriptional activation of lignin biosynthesis genes such as PAL, CCR, and CAD. These changes not only limit microbial access to intracellular substrates but also reduce the efficiency of fermentation, leading to weaker aroma profiles and less favorable texture in the final pickled products (Zhang et al., 2020). However, the traditional natural fermentation cycle is long, overly dependent on experience, not standardized, and far from being able to meet the surging industrial demand.

The main raw material for *suansun* is bamboo shoots. The traditional method for obtaining *suansun* is to peel, wash, and cut fresh bamboo shoots, soak them in mountain spring water or cold boiled water, and place them in a kimchi jar. Spontaneous fermentation at room temperature takes 15–30 days (Guan et al., 2020b). Direct inoculation with various fermentation agents is the main method used for making modern *suansun*. Screening functional strains can ensure the safety, stability, and flavor consistency of the product, and achieve targeted fermentation. *Lactobacillus plantarum* is one of the most commonly used strains in inoculation fermentation. It can effectively inhibit nitrite and pathogenic bacteria, while also enhancing the overall flavor of the product (Yu et al., 2023; Zhang et al., 2022). While pursuing production efficiency, maintaining the flavor of *suansun* is also a key focus of research. The structure of the microbial community of *suansun* varies depending on the region and fermentation method. The characteristic flavors of *suansun* include acidic substances such as acetic acid and lactic acid, *p*-cresol, and volatile substances such as valeraldehyde and ethyl acetate.

Electronic nose technology (E-nose) is a new type of artificial intelligence olfactory device for rapid and non-destructive testing, but it cannot separate and identify the aroma components of samples. Headspace solid-phase microextraction (HS-SPME) enables automation in the aroma enrichment process, with advantages such as convenience and speed. HS-SPME-gas chromatography-mass spectrometry (HS-SPME-GC-MS) is commonly used for detecting and identifying aroma components (Lin et al., 2013; Liu et al., 2020). SPME uses the chromatographic stationary phase on the surface of quartz fibers to extract components from the sample through adsorption for analysis. Unlike SPME, the adsorption layer of SBSE (stir bar adsorbent extraction) relies on van der Waals forces for the extraction of compounds, rather than surface adsorption. It not only requires lower thermal energy to release compounds, avoiding the loss of thermosensitive compounds, but also lacks surface competitiveness and has a larger linear range. Therefore, in this study, SBSE-GC-MS and electronic nose were used to systematically analyze the key flavors that affect direct fermentation and natural fermentation of *suansun*. An SVM algorithm was used to identify fermentation methods, and 16sRNA was used to explore the changes in bacterial structure during fermentation and the correlation with key flavor compounds.

## 2 Materials and methods

### 2.1 Sample preparation and collection of *suansun*

Fresh bamboo shoots of *Dendrocalamus latiflorus* harvested from Liucheng County, Liuzhou City, Guangxi Province were stored at 4°C and transported to the laboratory within 24 h. After removing the bamboo shoot shell, rinsing with sterile water, cutting it into 10 cm × 0.5 cm × 0.5 cm thin filaments, they were placed in ceramic jars with a capacity of 1 L. Each jar contained 800 g of bamboo shoot filaments, which were immersed in pure water (PWLS) and *Lactobacillus plantarum* solution (LP) for water sealed fermentation, as well as in pure water soaking without water sealed fermentation (PW). The inoculation amount of LP is 2% of the mass of bamboo shoots. Samples were taken on the 2nd, 8th, 14th, 20th, 32nd, and 38th days of fermentation, with three replicates for each sample, totaling 54 samples. Unopened samples were randomly selected for sterile sampling and stored at −80°C for further analysis.

*Lactobacillus plantarum* was purchased from Weihai Haixi (Shandong) Bioengineering Co., Ltd. Normal alkanes and 2,4,6-trimethylpyridine were purchased from Shanghai Anpu Company. The SBSE gas chromatography-mass spectrometer (8890-5977B) was from Agilent Technologies. The PEN3 portable electronic nose was from Airsense, a German company.

### 2.2 SBSE-GC-MS

#### 2.2.1 Extraction conditions for flavor compounds

Extracts from *suansun* samples stored at −80°C were obtained by physical extrusion using a round headed glass rod, and diluting 250 times with saturated saline solution. Then, 6 mL of the diluted solution was placed in a headspace vial along with Twisters that have been aged and 100 μL of internal standard substance (2,4,6-trimethylpyridine, 1 ng/mL), which was shaken and extracted at 50°C for 1.5 h. After extraction, the Twisters were rinsed with ultrapure water and the surface was cleaned with a dust-free tissue before placing them in a hot desorption sample tube for testing.

#### 2.2.2 MPS conditions

Thermal desorption (TDU) program: at a rate of 50°C/min, from an initial temperature of 10–250°C then held for 15 min without shunting; cold inlet (CIS) program: at a rate of 12°C/s, from an initial temperature of 10–250°C held for 10 min with a shunt ratio of 3:1.

#### 2.2.3 GC conditions

Agilent DB Wax capillary gas chromatography column (60 m × 0.25 mm × 0.25 μm), heating program: initial temperature of 40°C, held for 4 min; then raised to 140°C at a rate of 6°C/min, held for 5 min; then raised to 150°C at a rate of 3°C/min, held for 1 min; then raised to 197°C at a rate of 5°C/min and maintained for 2 min; then raised to 205°C at a rate of 1°C/min and maintained for 0 min; then raised to 240°C at a rate of 7°C/min and maintained for 15 min. The flow rate of the chromatographic column was 1.7 mL/min, with no split injections; the carrier gas was high-purity helium (He).

#### 2.2.4 MS conditions

Ionization method: EI, Electronic energy: 70 EV, transmission line temperature: 250°C, ion source temperature: 230°C, quadrupole temperature: 150°C, full scan mode (SCAN), scanning range: 25–250 da.

### 2.3 E-nose

Sample pretreatment: 5 g of solid *suansun* was weighed into a 100 mL beaker, sealed with cling film, and heated at a constant temperature water bath at 40°C for 10 min until the headspace gas reached equilibrium.

Test method: Under the conditions of a carrier gas flow rate of 400 mL/min, the carrier gas carried the sample headspace gas through the sensor array and into contact with the sensor array to generate a response signal. The sampling interval was 1 s, the signal detection time was set to 120 s, and the cleaning time was set to 60 s. Each sample was measured in parallel three times, and the average of the test data was taken for statistical analysis.

### 2.4 DNA extraction

Total community genomic DNA was extracted using the E.Z.N.A™ MagBind Soil DNA Kit (M5635-02, Omega Bio-Tek, Norcross, GA, USA) following the manufacturer’s instructions. The DNA concentration was quantified using a Qubit 4.0 fluorometer (Thermo Fisher Scientific, Waltham, MA, USA) to ensure that adequate amounts of high-quality genomic DNA had been extracted.

### 2.5 16S rRNA gene amplification

The target was the V3–V4 hypervariable region of the bacterial 16S rRNA gene. The region was amplified by PCR immediately upon DNA extraction. The 16S rRNA V3–V4 fragment was amplified using two universal bacterial 16S rRNA gene amplicon PCR primers (PAGE purified): the amplicon PCR forward primer (CCTACGGGNGGCWGCAG) and amplicon PCR reverse primer (GACTACHVGGGTATCTAATCC). The reaction mixture composition was as follows: 2 μL microbial DNA (10 ng/μl), 1 μL amplicon PCR forward primer (10 μM), 1 μL amplicon PCR reverse primer (10 μM), and 30 μL 2 × Hieff®Robust PCR Master Mix (Yeasen, 10105ES03, Shanghai, China). The plate was sealed and the PCR performed in a thermal-cycling instrument (Applied Biosystems 9700, Foster City, CA, USA) using the following program: 1 cycle of denaturation at 95°C for 3 min; 5 cycles of denaturation at 95°C for 30 s, annealing at 45°C for 30 s, and elongation at 72°C for 30 s; 20 cycles of denaturation at 95°C for 30 s, annealing at 55°C for 30 s, and elongation at 72°C for 30 s; and a final extension at 72°C for 5 min. The PCR products were checked by electrophoresis in 2% (w/v) agarose gels in TBE buffer stained with ethidium bromide and visualized under ultraviolet light.

### 2.6 16S gene library construction, quantification, and sequencing

Hieff NGS™ DNA Selection Beads (Yeasen, 10105ES03) were used to purify the free primers and primer dimer species in the amplicon product. Samples were delivered to Sangon BioTech (Shanghai, China) for cDNA library construction using universal Illumina adaptors and indices. Before sequencing, the DNA concentration of each PCR product was determined using a Qubit 4.0 Green double-stranded DNA assay and quality control was performed with a bioanalyzer (Agilent 2100, Santa Clara, CA, USA). Depending on the coverage requirement, all libraries could be pooled for one run. The amplicons from each reaction mixture were pooled in equimolar ratios based on their concentration. Sequencing was performed using the Illumina MiSeq system (Illumina, San Diego, CA, USA) in accordance with the manufacturer’s instructions.

### 2.7 Sequence processing, OTU clustering, representative tags alignment, and taxonomic classification

After sequencing, the two short-read Illumina datasets were assembled with PEAR software (version 0.9.8) based on overlap in the short reads. The FASTQ files were processed to generate individual FASTA and QUAL files, which could then be analyzed by standard methods. The effective tags were clustered into operational taxonomic units (OTUs) of ≥97% similarity using Usearch software (version 11.0.667). Chimeric sequences and singleton OTUs (with only one read) were removed, after which the remaining sequences were sorted into each sample based on the OTUs. The tag sequence with the highest abundance was selected as a representative sequence within each cluster. Bacterial and fungal OTU representative sequences were classified taxonomically by conducting a BLAST search against the RDP database and the UNITE database, respectively.

### 2.8 Statistical analysis

Partial least squares discriminant analysis was performed using SIMCA 14.1 software and SVM discriminant analysis was performed in R language. Qualitative analysis: NIST23 spectral library and Aroma Office 2D flavor library were used to search and compare, combined with the retention index (RI) for qualitative analysis, screening compounds with a match of more than 70, and at the same time, a mixture of C7–C40 n-alkanes was injected into the sample individually, and the warming procedure was consistent with the conditions of the GC-MS assay, so as to calculate the RI of each volatile compound. The RI of each volatile was calculated and compared with the RI values in the literature. Quantitative analysis: Using 2,4,6-trimethylpyridine as internal standard, the content of each component in the sample was calculated based on the concentration of the internal standard and the ratio of the peak area of each component in the sample to the peak area of the internal standard.


(1) $$\mathrm{RI}=100\times (n+\frac{t_{i}-t_{n}}{t_{n+1}-t_{n}})$$

RI is the retention index; n is the number of carbons; t_i_ is the retention time of the volatile component to be measured; t_n_ is the retention time of the n-alkanes; t_n + 1_ is the retention time of the (n + 1) carbon n-alkanes.


(2) $$C_{1}=\frac{A_{1}\times C_{0}\times V_{0}}{A_{0}\times V_{1}}$$

C_1_ is the concentration of the compound to be tested (μg/mL), C_0_ is the concentration of the internal standard (2,4,6-trimethylpyridine; μg/mL), A_1_ is the peak area of the compound to be tested, A_0_ is the peak area of the internal standard, V_1_ is the volume of the extracted sample (i.e., 6.0 mL), and V_0_ is the volume of the internal standard (i.e., 100 μL).

To identify key aroma compounds that contributed to the differentiation among fermentation methods, partial least squares discriminant analysis (PLS-DA) was conducted using SIMCA 14.1 software. Compounds were selected as potential discriminative markers if they met two criteria: (1) a variable importance in projection (VIP) score greater than 1.0 from the PLS-DA model, indicating a strong contribution to sample separation, and (2) a statistically significant difference among groups (*p* < 0.05), as determined by one-way ANOVA.

## 3 Results and discussion

### 3.1 Analysis of quality differences in *suansun* based on E-nose technology

**Figure 1:**
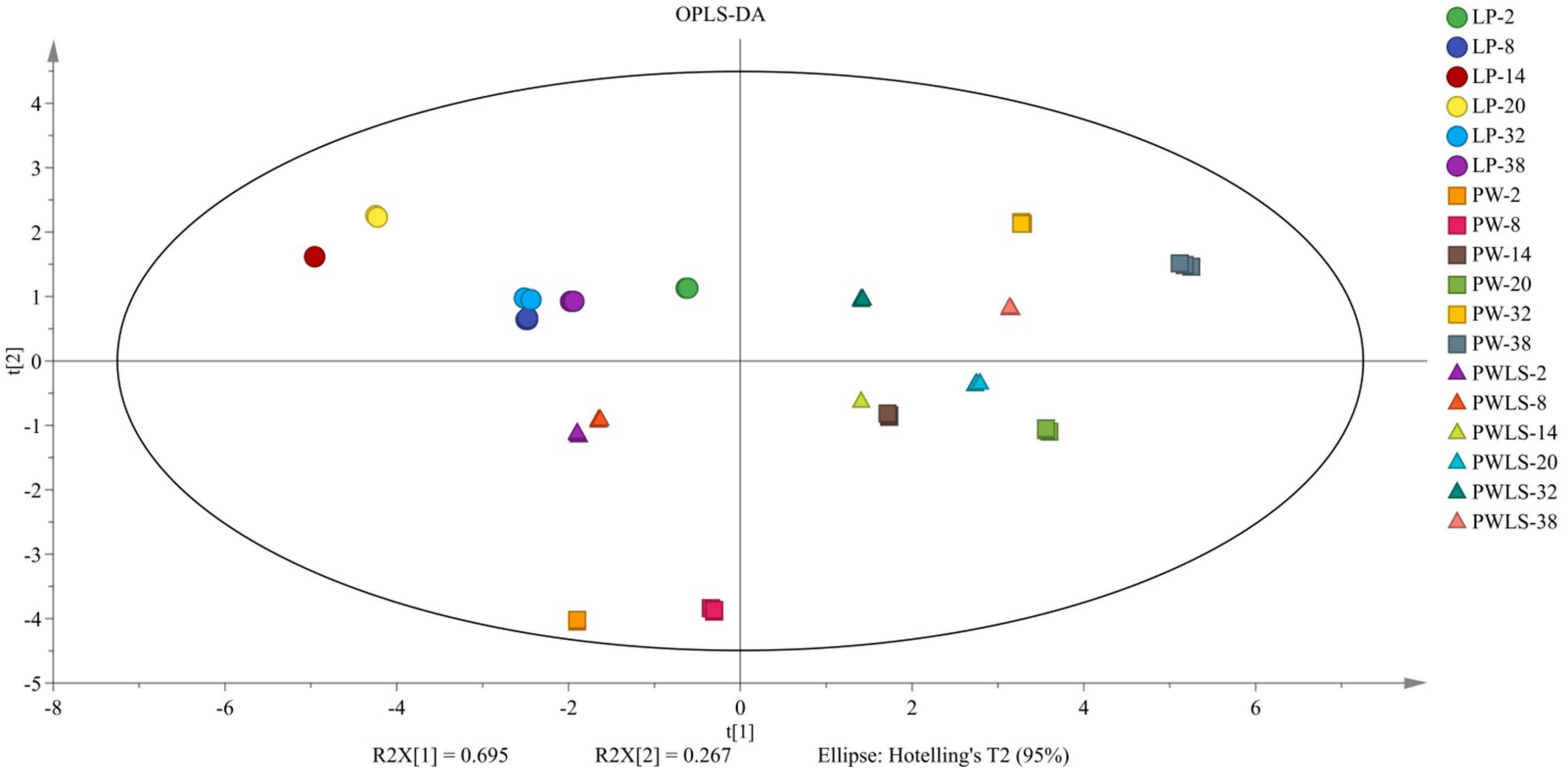
OPLS-DA scatter plot of *suansun* electronic nose treated with different fermentation methods.

As shown in Figure 1, the *suansun* treated with three different fermentation methods were separated on both the horizontal and vertical axes of the score scatter plot, with an independent variable fitting index (R^2^X) of 1 and a dependent variable fitting index (R^2^X) of 0.568. The LP scores throughout the fermentation process were concentrated in one quadrant, achieving a clear distinction from the PW and PWLS treatments. As fermentation progressed, PW and PWLS were divided into three groups in the horizontal and vertical axes, namely on the 2nd and 8th days, 14th and 20th days, and 32nd and 38th days of fermentation, evenly distributed in the other three quadrants. Adding *Lactobacillus plantarum* stabilized the fermentation flavor of *suansun* without significant fluctuations due to prolonged fermentation time. In summary, there is a clear distinction between direct fermentation and natural fermentation in both the horizontal and vertical axes.

**Figure 2:**
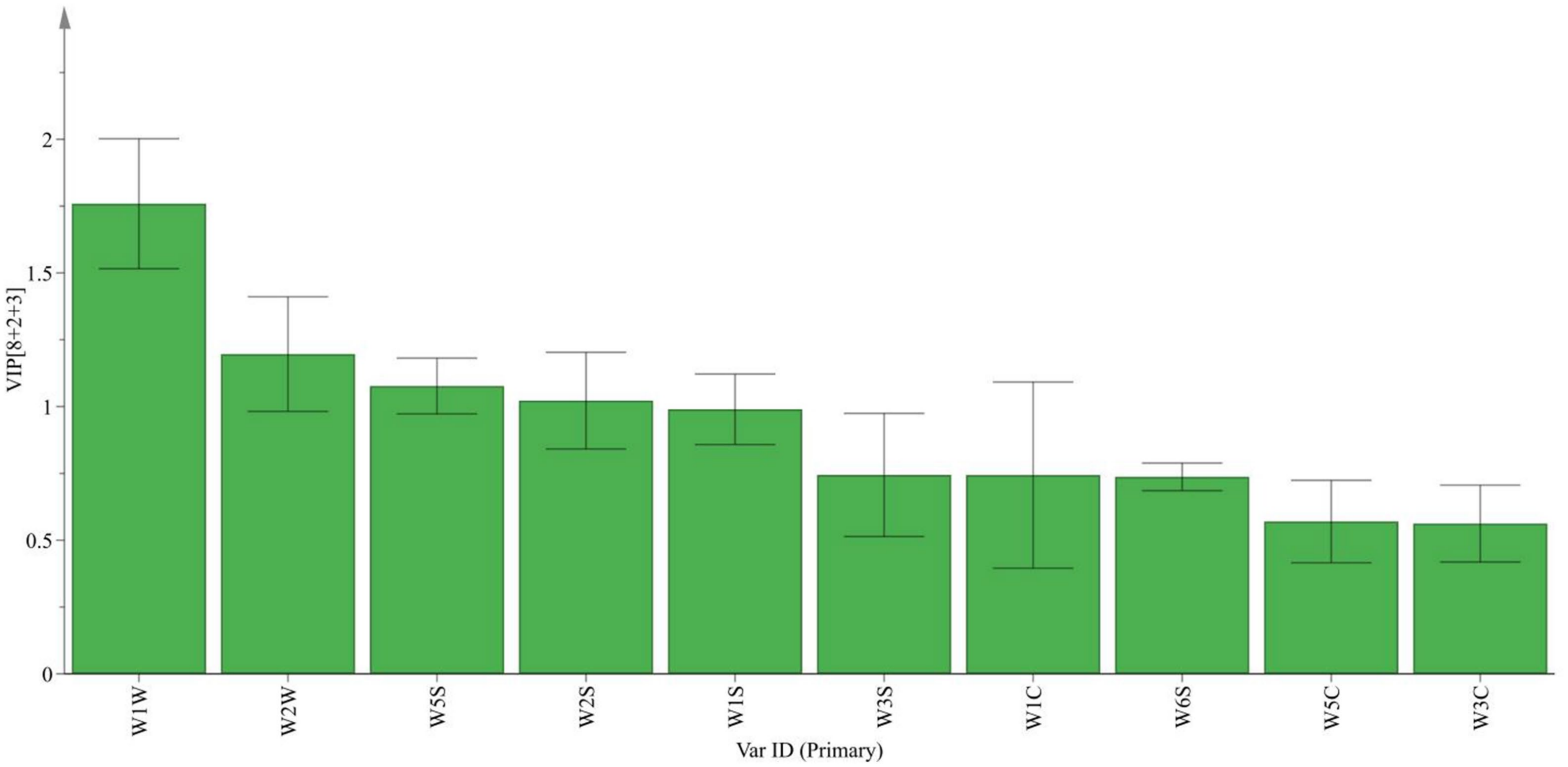
VIP of *suansun* electronic nose treated with different fermentation methods.

**Figure 3:**
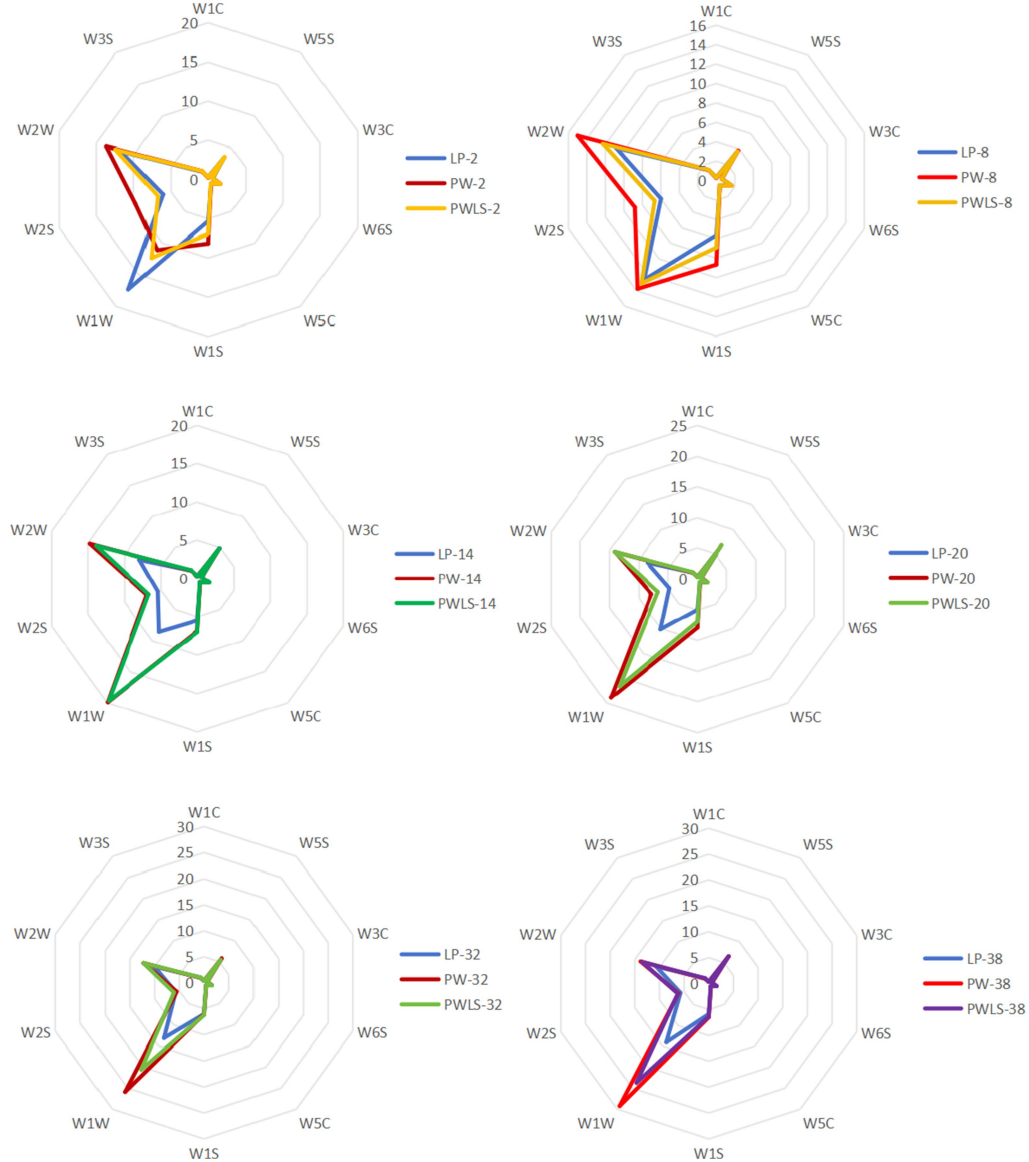
Radar chart of *suansun* electronic nose treated with different fermentation methods.

Based on the data in Table 1, a VIP map of electronic nose characteristic odor (Figure 2) and a radar fingerprint map (Figure 3) were plotted. It can be seen that the response values of 10 sensors to the volatile components of *suansun* obtained by three different fermentation methods are different. Among the 10 sensors, W1W, W2W, W5S, W2S, and W1S made significant contributions to the OPLS-DA model in this experiment. Among them, W1W was sensitive to sulfides, and on the second day of fermentation, the response value of W1W sensor to direct injection fermentation was significantly higher than that of the two natural fermentations. When a watershed appeared on the 8th day of fermentation, the response value of W1W sensor to LP was slightly lower than that of PW and PWLS. As fermentation continued, the response value of LP was significantly lower than that of PW and PWLS. In addition, there was no significant difference in the response values of WIW to PW and PWLS, and from the 8th day of fermentation, the response values of PW were slightly higher than those of PWLS. Sensor W2S was sensitive to alcohols, aldehydes, and ketones, and the response value of LP is significantly lower than PW. As fermentation progressed, the gap continued to narrow until the response was comparable on the 38th day of fermentation. Overall, the response values of LP to the five key sensors were lower or comparable to PW and PWLS. The difference between PW and PWLS was mainly reflected in the first 8 days of fermentation, with significant differences in response to three sensors: W1S, W2S, and W2W. W1S was sensitive to methyl groups, while W2W was sensitive to aromatic components and organic sulfides. After 14 days of fermentation, PW and PWLS had comparable responses to the five sensors.

**Table 1:** Electronic nose response values for *Suansun* treated with different fermentation methods (partial).

Array serial number	Sensor name	Sensors respond to sensitive substances	Response value		
			LP-38	PW-38	PWLS-38
1	W1C	Aromatic components, benzene derivatives	0.35 ± 0.00023	0.34 ± 0.0011	0.34 ± 0.00046
2	W5S	Nitrogen oxides, high sensitivity	3.67 ± 0.0039	6.53 ± 0.044	6.46 ± 0.0062
3	W3C	Aromatic components, ammonia compounds	0.63 ± 0.00026	0.69 ± 0.00069	0.64 ± 0.00083
4	W6S	Hydrogenated compounds, hydrogen gas	1.56 ± 0.00035	1.53 ± 0.00050	1.59 ± 0.00042
5	W5C	Aromatic components of short chain alkanes	0.65 ± 0.0035	0.71 ± 0.0017	0.65 ± 0.00035
6	W1S	Methyl group	5.86 ± 0.0019	6.47 ± 0.018	6.31 ± 0.0056
7	W1W	sulfide	13.93 ± 0.031	29.23 ± 0.11	23.69 ± 0.0048
8	W2S	Alcohols, aldehydes and ketones	5.73 ± 0.00052	6.15 ± 0.029	6.26 ± 0.0055
9	W2W	Aromatic components, organic sulfides	10.69 ± 0.0038	13.82 ± 0.059	13.51 ± 0.0099
10	W3S	Long chain alkanes	1.29 ± 0.00025	1.15 ± 0.00046	1.27 ± 0.00064

### 3.2 Analysis of aroma component characteristics

**Figure 4:**
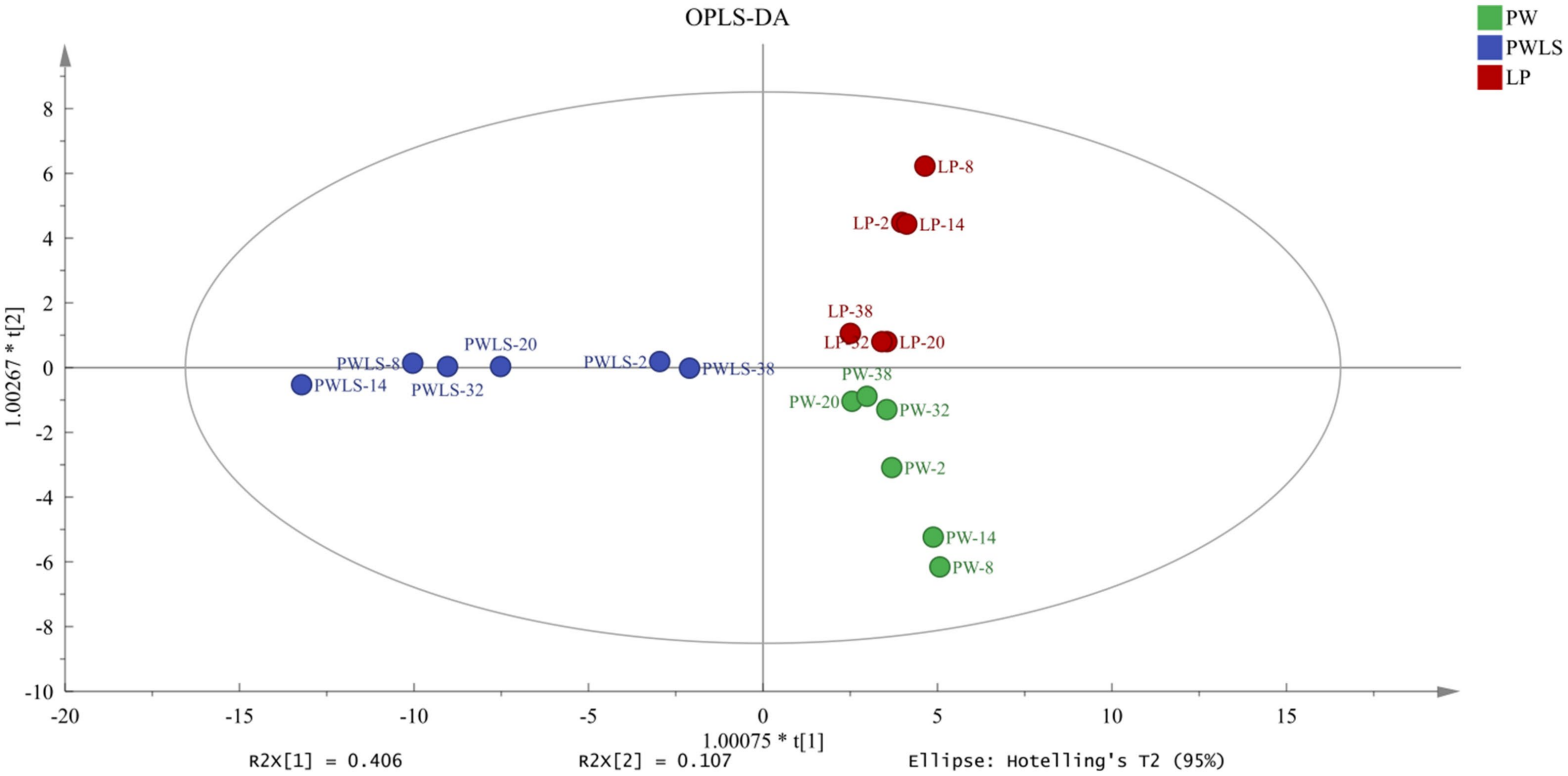
OPLS-DA plots of *suansun* treated with different fermentation methods.

**Figure 5:**
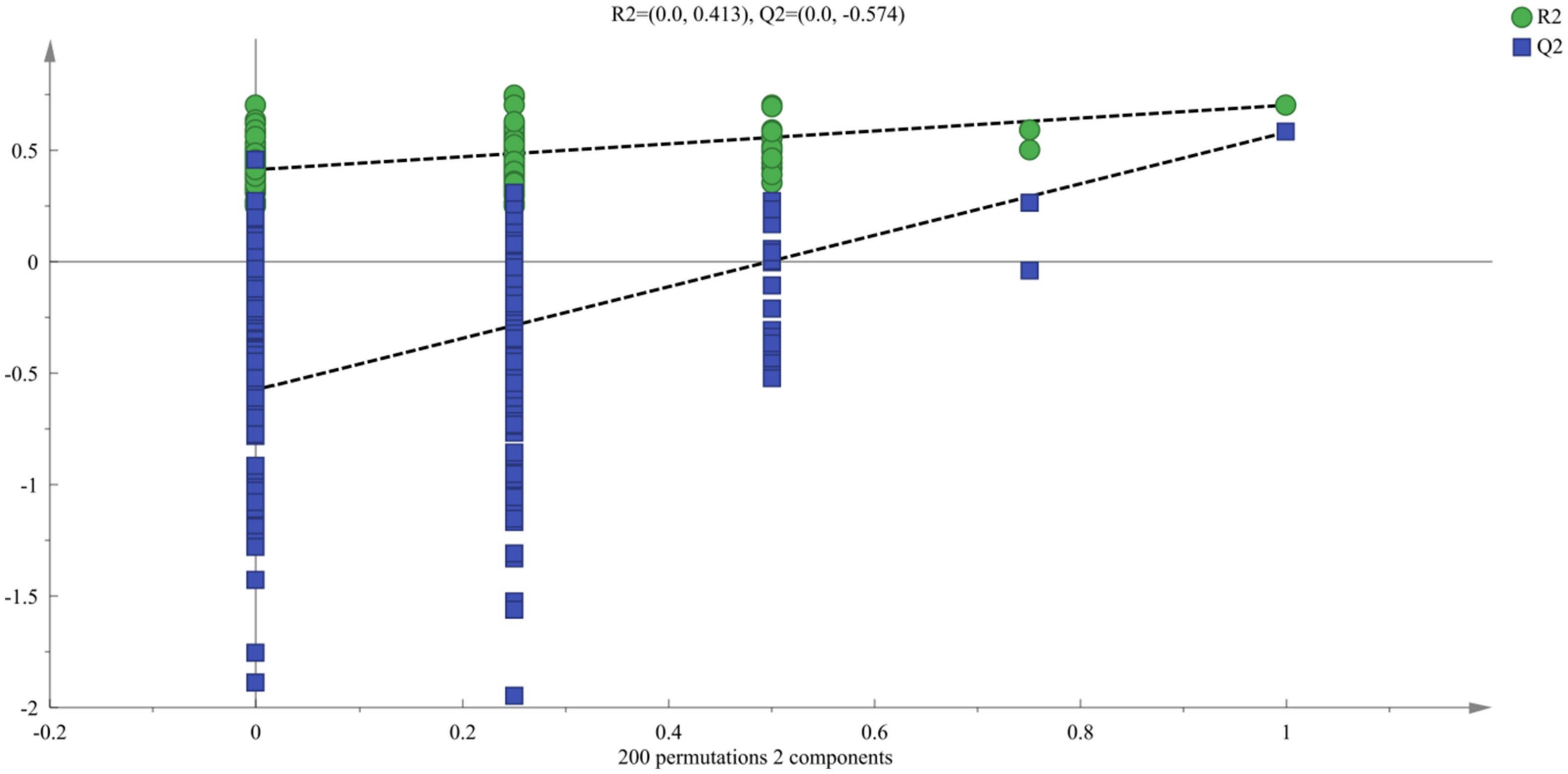
Permutation test.

To study the changes in aroma of *suansun* produced by the three different fermentation methods during the fermentation process, SBSE-GC-MS was used to analyze and identify the aroma components and contents of 54 *suansun* samples, and a total of 148 common aroma components were detected. Using 148 common aroma components as dependent variables and different fermentation methods as independent variables, OPLS-DA (Figure 4) can effectively distinguish different fermentation methods. The independent variable fitting index (R^2^x) in this analysis was 0.884, the dependent variable fitting index (R^2^y) was 0.746, and the model prediction index (Q^2^) was 0.617. If R^2^ and Q^2^ exceeded 0.5, the model fitting results were acceptable. After 200 replacement tests, as shown in Figure 5, the intersection point between the Q^2^ regression line and the vertical axis was less than 0, indicating that the model did not exhibit overfitting and the model validation was effective. It is believed that this method can be used for the identification and analysis of aroma components in *suansun* treated with different fermentation methods.

**Figure 6:**
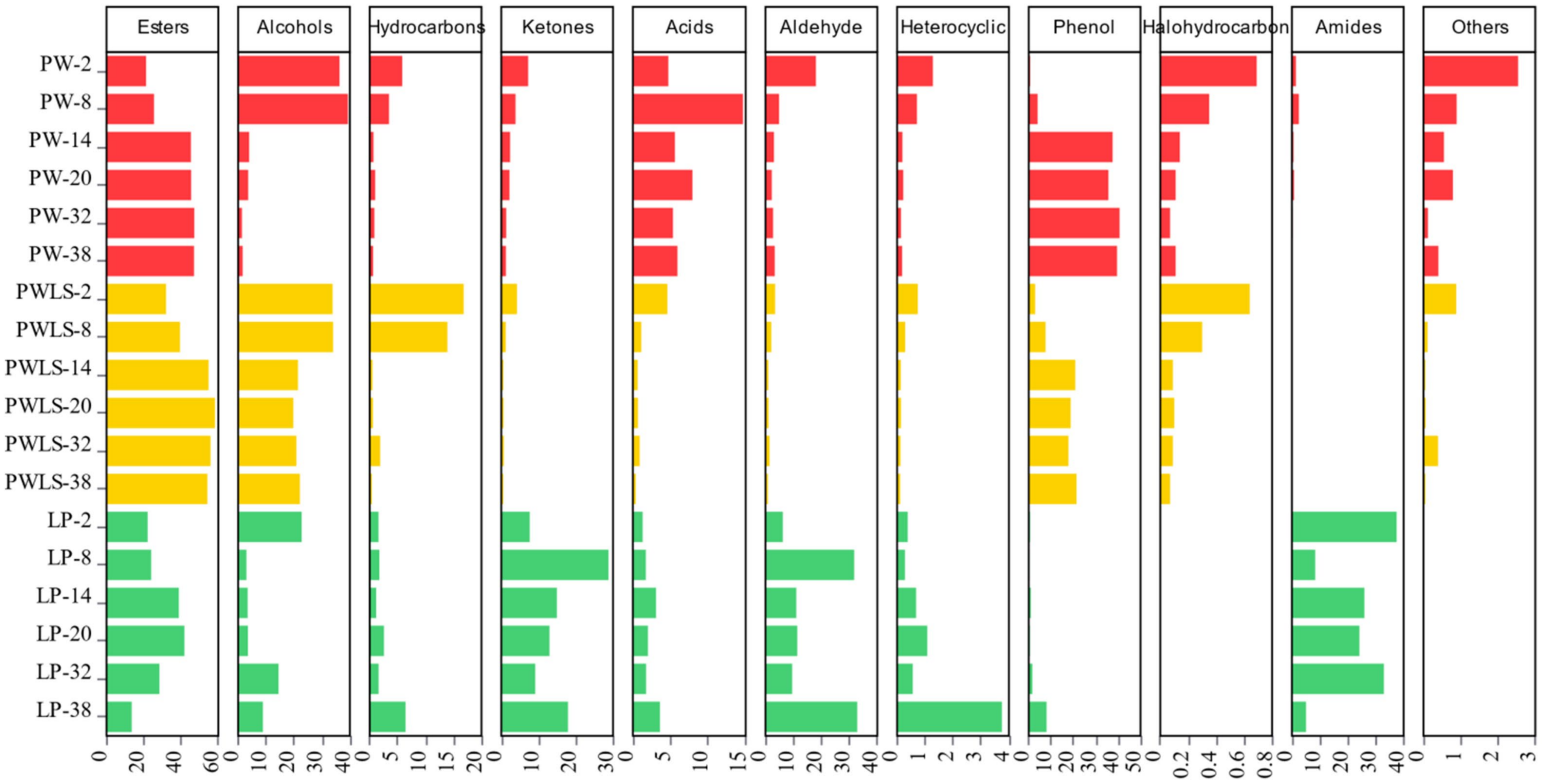
Bar chart of 11 flavor compounds in *suansun* treated with different fermentation methods.

Based on 148 common aroma components, further removal of substances that did not have any more information in the NIST 23.LT and Aroma Office 2D flavor libraries (Equations 1, 2) resulted in the screening of 114 compounds (as shown in Figure 6), including up to 31 esters, 16 alcohols, 16 hydrocarbons, 16 ketones, 9 acids, 7 aldehydes, 6 heterocyclic compounds, 4 phenols, 4 halogenated hydrocarbons, 3 amides, and 2 others. The main volatile flavor compounds in the early stage of fermentation (the 2nd day of fermentation) using two natural fermentation methods were alcohols, consistent with existing literature reports (Guan, 2023). Alcohol compounds account for 36.29 and 33.8% of the total volatile substance content in PW and PWLS, respectively. As fermentation continued, esters, alcohols, and phenols were the main volatile flavor compounds in the later stages of fermentation, consistent with the sensitivity of W2S, one of the five sensors that contributed significantly to electronic nose detection, to alcohols, aldehydes, and ketones. From the 14th to the 38th day of fermentation, the main flavor compounds in PW remained stable, mainly esters (45.32–46.98%) and phenols (35.65–40.66%), while PWLS mainly consisted of esters (54.14–58.19%), alcohols (19.77–22.11%), and phenols (17.78–21.38%). Direct fermentation was completely different from natural fermentation. Amides (37.49%) were the main flavor compounds in the early stages of LP, while esters, ketones, aldehydes, and amides were the main compounds in the later stages of fermentation. Direct fermentation consistently exhibited high levels of esters and aldehydes, as well as low levels of phenols, through the Ehrlich route, which *Lactobacillus plantarum* uses to transform free amino acids in *suansun* into aldehydes (Xue et al., 2025). By using L-histidine and L-phenylalanine, this procedure lessens astringency and bitterness (Cai et al., 2019). In summary, starting from 14 days of natural fermentation, there is a significant change in the main categories of flavor compounds, such as esters, alcohols, and phenols, with a noticeable increase or decrease in their content. This is consistent with the results from the electronic nose, which showed significant changes in response after 14 days of fermentation, an important time point for natural fermentation. The *p*-cresol in phenolic compounds is the main characteristic flavor substance in *suansun*, with a unique “odor” (Guo et al., 2021). In natural fermentation samples, *p*-cresol reached its peak on the 32nd day, while PW samples increased from 0.0065 μg/mL to 1.24 μg/mL, with a content increase of 190.77 times. The PWLS sample increased from 0.017 μg/mL to 10.22 μg/mL, which was also the flavor substance with the highest content detected. The results were consistent with the literature (Li et al., 2022), with a content increase of 601.17 times. It is worth noting that on the second day of direct fermentation, the highest concentration of *p*-cresol was 0.023 μg/mL. As fermentation progressed, the concentration gradually decreased to 0.0022 μg/mL, a decrease of 10.45 times. From the 8th day onwards, the *p*-cresol content was significantly lower than that of natural fermentation. Guan et al. collected 50 samples of bamboo shoots from 23 regions in Guangxi, Guangdong, Yunnan, and Fujian provinces and found that the content of *p-creso*l increased by 64.5 times throughout the fermentation process (Guan, 2023).

The pronounced accumulation of p-cresol observed during the water-sealed fermentation (PWLS) is likely attributable to enhanced microbial tyrosine metabolism under strictly anaerobic conditions. Previous studies have identified tyrosine metabolism as one of the most enriched functional pathways in naturally fermented bamboo shoots (Yu et al., 2006), underscoring its pivotal role in amino acid transformation during fermentation. In this pathway, tyrosine is first converted to p-hydroxyphenylpyruvate via tyrosine aminotransferase, followed by decarboxylation to p-hydroxyphenylacetate and subsequent conversion to p-cresol, catalyzed by p-hydroxyphenylacetate decarboxylase (4-HPA decarboxylase), as reported in *Clostridium difficile* (Yu et al., 2006). This enzymatic process is highly oxygen-sensitive and is typically activated under anaerobic conditions.

Metagenomic annotations have further confirmed the presence of key genes encoding these enzymes in *Lactiplantibacillus plantarum* and *Lacticaseibacillus* fermentum, both of which were found to dominate the later stages of PWLS. In addition, recent studies have suggested an alternative route for p-cresol biosynthesis via S-adenosylmethionine (SAM)-dependent methylation of tyrosine in certain lactic acid bacteria (Chen et al., 2023). Collectively, these findings provide a mechanistic basis for the observed 600-fold surge in p-cresol under PWLS and highlight the synergistic effects of microbial composition and the unique anaerobic environment on phenolic compound accumulation.

### 3.3 Analysis of differences in aroma components of different *suansun*

**Figure 7:**
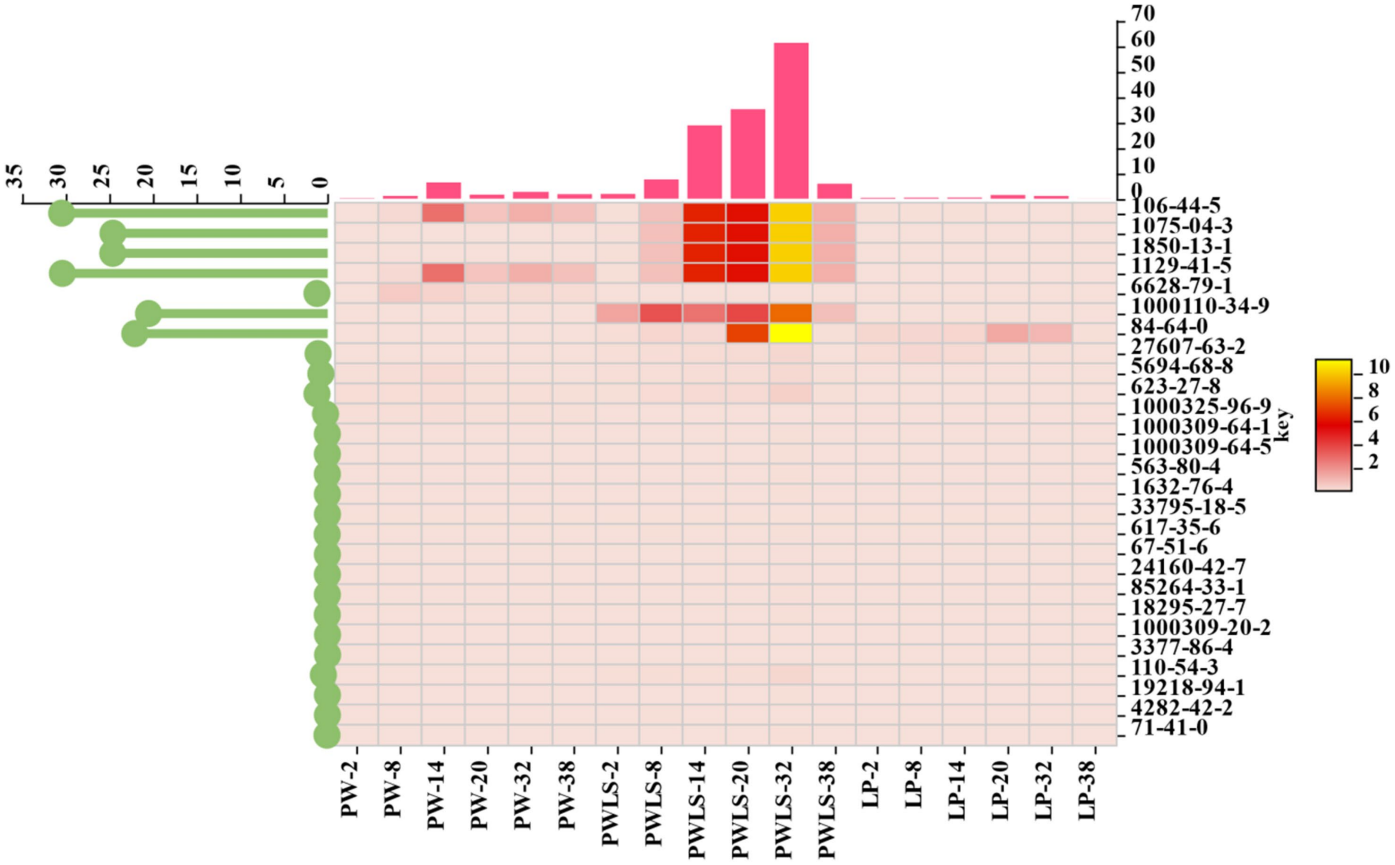
Thermogram of different aroma composition combinations of *suansun* processed by different fermentation methods.

In order to further analyze the contribution of different aroma components to distinguishing different fermentation methods, 27 different aroma substances from three different fermentation methods were screened based on the criteria of *p* < 0.05 and VIP > 1 (Figure 7), including 7 esters, 5 alcohols, 4 halogenated hydrocarbons, 3 heterocyclic compounds, 2 acids, 2 ketones, 1 phenol, 1 aldehyde, 1 hydrocarbon, and 1 sulfonate ester. Please refer to Table 2 for details. From the lollipop chart on the left side of the heatmap in Figure 7, it can be seen that 6 out of the 27 key flavor compounds showed outstanding performance, namely *p*-cresol, 1-phenylpropane-1,2-diol, carbamic acid, p-tolyl ester, 3-methylphenyl-N-met, 1,2-diphenylethanol, 1,2-benzenedicarboxylicacid, 1-butyl 2-cyclohexyl ester. Throughout the fermentation period (2–38 days), three key flavor substances, 1-phenylpropane-1,2-diol, 1,2-diphenylethanol, and carbamic acid, *p*-tolyl ester, were present only in PWLS. The bar graph above the heat map shows the sum of the variables in each column, showing that the sum of the key core flavor components in PWLS was significantly different from the other two treatments, reaching its highest value at day 32 of fermentation. Throughout the metabolic processes of microorganisms, macromolecular compounds were oxidized and hydrolyzed during *suansun* fermentation, producing a high number of taste precursors and causing a rapid increase in volatile chemicals (Raghuvanshi et al., 2019).

**Table 2:** 27 different aroma substances.

No	Category	CAS No	Compound name	Formula
1	Esters	1850-13-1	Carbamic acid-*p*-tolyl ester	C_8_H_9_NO_2_
2	Esters	1129-41-5	3-Methylphenyl-*N*-met	C_9_H_11_NO_2_
3	Esters	1000309-64-1	Oxalic acid, monoamide, n-propyl, propyl ester	C_8_H_15_NO_3_
4	Esters	1000309-64-5	Oxalic acid, monoamide, n-propyl, hexyl ester	C_11_H_21_NO_3_
5	Esters	617-35-6	Pyruvic acid ethyl ester	C_5_H_8_O_3_
6	Esters	1000325-96-9	(3*R*,6*S*)-3,6-Dimethyl-2,4,5,6-tetrahydropyrimidine	C_18_H_17_ClO_4_
7	Esters	84-64-0	1,2-Benzenedicarboxylicacid-1-butyl 2-cyclohexyl ester	C_18_H_24_O_4_
8	Alcohols	1075-04-3	1-phenylpropane-1,2-diol	C_9_H_12_O_2_
9	Alcohols	5694-68-8	1,3-Dioxolane-2-methanol	C_4_H_8_O_3_
10	Alcohols	85264-33-1	1H-Pyrazole-1-methanol,3,5-dimethyl-	C_6_H_10_N_2_O
11	Alcohols	71-41-0	n-Amyl alcohol	C_5_H_12_O
12	Alcohols	1000110-34-9	1,2-Diphenylethanol	C_18_H_20_O
13	Halogenated Hydrocarbons	18295-27-7	Butane, 2-iodo-3-methyl-	C_5_H_11_I
14	Halogenated Hydrocarbons	3377-86-4	Hexane, 2-bromo-	C_6_H_13_Br
15	Halogenated Hydrocarbons	4282-42-2	1-Iodononane	C_9_H_19_I
16	Halogenated Hydrocarbons	19218-94-1	Tetradecane, 1-iodo-	C_14_H_29_I
17	Heterocyclic Compounds	1632-76-4	3-Methylpyridazine	C_5_H_6_N_2_
18	Heterocyclic Compounds	67-51-6	3,5-Dimethylpyrazole	C_5_H_8_N_2_
19	Heterocyclic Compounds	24160-42-7	β-Methyl-1H-imidazole-4-ethanamine	C_6_H_11_N_3_
20	Acids	6628-79-1	Pentanoic acid-3-methyl-4-oxo-	C_6_H_10_O_3_
21	Acids	33795-18-5	Phosphonic acid-*p*-(4-hydroxyphenyl)-	C_6_H_7_O_4_P
22	Ketones	27607-63-2	5-Ethyl-4-methyl-3-heptanone	C_10_H_20_O
23	Ketones	563-80-4	3-Methyl-2-butanone	C_5_H_10_O
24	Phenols	106-44-5	*p*-Cresol	C_7_H_8_O
25	Aldehydes	623-27-8	1,4-Phthalaldehyde	C_8_H_6_O_2_
26	Hydrocarbons	110-54-3	Hexane	C_6_H_14_
27	Sulfonate Esters	1000309-20-2	Propanoic acid, 2-methyl-3-hydroxy-2,2,4-trimethylpentyl ester	C_14_H_30_O_3_S

### 3.4 Discriminant analysis of fermentation mode based on differential aroma components

**Figure 8:**
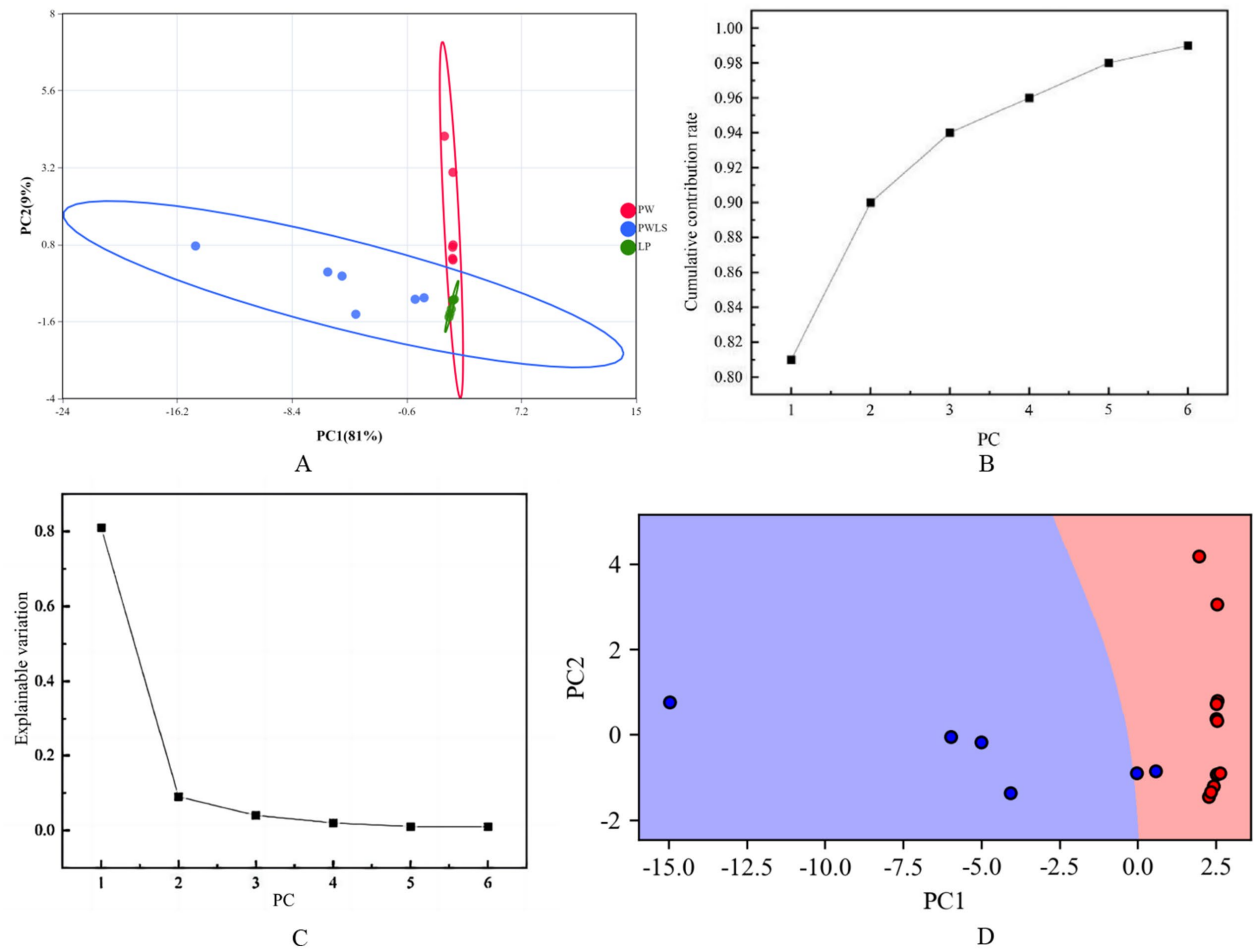
PCA plot of differential aroma of *suansun* treated with different fermentation methods. **(A)** Scatter plot of suansun scores for the first two PCs. **(B)** Cumulative variance ratio of the first two PCs. **(C)** Explained variance of each principal component. **(D)** SVM model discrimination rate using the first two PCs.

In order to obtain more accurate and intuitive classification results, based on the 27 aroma components screened with intergroup differences, the aroma data of *suansun* treated with three different fermentation methods were analyzed by fermentation method discrimination. Differential aroma data were downscaled using PCA to obtain scatter plots of *suansun* scores for different fermentation treatments for the first 2 PCs (Figure 8A). As shown in Figure 8B, the closer the PC cumulative ratio to 1, the more reliable the PCA model. As shown in Figure 8C, the explainable variance of each PC is PC1 > PC2 > PC3 > PC4 > PC5 > PC6, in which the variance contribution rates of PC1 and PC2 are 81 and 9% respectively, and the cumulative variance contribution rate reaches 90%, which demonstrates that the artificial selection of PC1 and PC2 analysis samples has good reliability. The SVM model was based on the first 2 PCs, and the samples were randomly divided into a training set (70%) and a test set (30%), and the SVM model discrimination rate reached 100% (Figure 8D).

### 3.5 Association analysis of dominant colonies with characteristic flavor substances

**Figure 9:**
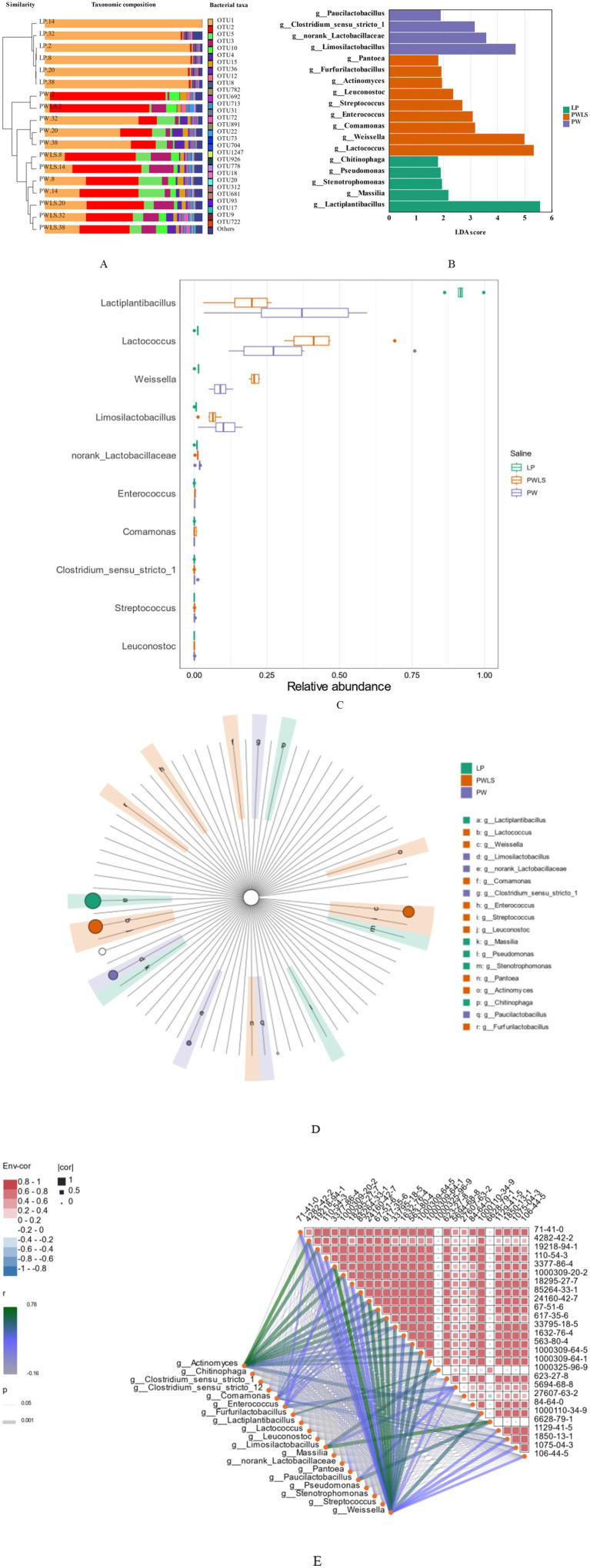
**(A)** Bacterial community structure of different *suansun* at the genus level; **(B–D)** LDA value distribution bar chart, abundance difference chart, and classification tree chart of different bacterial colonies in *suansun* at the genus level; **(E)** Correlation network heatmap between 19 dominant bacterial genera and 27 key flavor compounds.

Figure 9A shows the structure of the three acid shoot bacterial communities at the genus level (only the top 30 bacteria in terms of mean relative abundance are listed; other bacterial genera are denoted by “other”). The sum of the abundances of the genera listed in the figure accounted for more than 94.2% of the total bacterial genus abundance in each sample, which reflects the main genus information of each sample. Some researchers have designated genera with average relative abundance ≥1% as dominant genera (Tang et al., 2022, 2024). *Lactiplantibacillus* was the bacterial genus with the highest mean relative abundance (48.52%) in the *suansun* samples, with a range of relative abundance from 3.04 to 99.69% in the different samples, and was the dominant genus in all samples. This is inconsistent with some reported findings, which showed that the dominant genus was *Lactobacillus* (Darmayanti et al., 2014; Guan et al., 2020a; Thakur et al., 2015). This may be due to the renaming of the bacterial genus. In 2020, the International Commission on Bacteriological Nomenclature undertook a comprehensive review and update of the classification of wild forking bacteria. Among the most notable changes was the renaming of a portion of the genus *Lactobacillus* as *Lactiplantibacillus*. The reclassification of *Lactobacillus plantarum* to *Lactiplantibacillus plantarum* was part of a comprehensive taxonomic revision based on whole-genome sequencing (Zheng et al., 2020). This study systematically reassigned multiple plant-associated strains, including *L. plantarum*, into the newly proposed genus *Lactiplantibacillus*. Earlier literature (Luo et al., 2020; Raghuvanshi et al., 2019; Romi et al., 2015; Thakur et al., 2015) still refer to *Lactobacillus plantarum*, whereas more recent research (Botthoulath et al., 2024; Jian et al., 2024; Qian et al., 2024; Qin et al., 2024; Wu et al., 2025; Xue et al., 2025) employs the updated *Lactiplantibacillus* nomenclature. Despite the change in genus name, functional roles such as acid production, amino acid metabolism, and flavor compound generation remain highly consistent across both nomenclatures. Therefore, we believe our results align with both legacy and current studies, and adopting the updated classification enhances consistency with contemporary microbial ecology frameworks. Similar to most vegetable fermentations, the fermentation process of *suansun* is significantly influenced by lactic acid bacteria (Das et al., 2023). The main lactic acid bacteria strain contained *Weissella* sp., *Lactobacillus plantarum*, *Lactobacillus curvatus*, *Pediococcus pentosaceus, and Leuconostoc* sp., (Chen et al., 2022; Guan et al., 2020b; Li et al., 2023).

The abundance of *Lactiplantibacillus* in LP increased rapidly on day 2 of fermentation (92.03% relative abundance) and peaked on fermentation day 14 (99.69% relative abundance), which was significantly higher than that of the PW and PWLS treatment groups, and then began to decline gradually to a minimum on day 32 of fermentation (86.17% relative abundance). Similar to the findings of Botthoulath et al. (2024) who used *Lactiplantibacillus plantarum* BBS13 as a fermenter, the strain counts reached a maximum at approximately 9 log CFU/g during the first 3 days of fermentation, with a sharp decline occurring between day 7 and the end of fermentation (Botthoulath et al., 2024). Xue et al. showed that, in the inoculation of *Lactobacillus plantarum* DACN768-fermented *suansun*, the content of 56 volatile flavor compounds peaked at 14 d (Xue et al., 2025). The natural fermentation, on the other hand, started to show a sharp increase in flavor content only on day 14, which peaked on fermentation day 32. This suggests that inoculation with *Lactobacillus plantarum* can improve on the effects of the natural microflora present in the raw material and has the potential to greatly enhance the production cycle. In addition, this study also found that *Lactococcus* (24.86%), *Weissella* (7.68%), and *Limosilobacillus* (5.52%) have relatively high average relative abundance, making them the dominant bacterial genera in *suansun*. Similarly, water sealed fermentation is used, but *Lactobacillus* and *Lactobacillus* were dominant at the genetic level in *suansun* soaked in mountain spring water (Chen et al., 2022). *Weissella*, *Lactococcus*, and *Lactobacillus* were the predominant genera in the fermentation process, as demonstrated by the natural fermentation of *suansun* (Romi et al., 2015). These are similar to our natural water-sealed fermentation results. There are few reports on *Limonolobacillus* as the dominant bacterial genus in naturally fermented (non-water sealed) *suansun*, and this difference may be caused by factors such as sampling location, *suansun* production process, and fermentation time (Jian et al., 2024; Tang et al., 2024).

Figure 9B shows the characteristics of microorganisms at different taxonomic levels under various fermentation conditions, and identifies biomarkers with significant differences between different *suansun* samples through linear discriminant analysis effect size (LEfSe) analysis. Overall, the linear discriminant analysis score (LDA) scores of different bacterial genera in different samples showed a clear discrete distribution, indicating significant differences in microbial community composition among different samples. Each sample had unique distribution characteristics of bacterial genera, and a total of 18 dominant bacterial genera were identified. In the LP fermentation system, *Lactiplantibacillus*, which may have been added due to the advantage of direct injection fermentation, can reproduce in large numbers and become the dominant microbial community, with an abundance range of 86.17–99.69%, resulting in a high LDA value and a single bacterial species composition, becoming a key microbial marker for distinguishing other samples. In the PWLS and PW fermentation systems, the dominant bacterial genera include *Lactococcus*, *Weissella*, and *Limosilobacillus*. The selected biomarkers include not only bacterial genera with high average relative abundance mentioned above, but also *Clostridiumsensustricto1* with an average relative abundance of 0.008%. Figure 9C provides a visual representation of the differences in average relative abundance of different bacterial genera in the various fermentation systems. The classification tree diagram in Figure 9D shows the hierarchical classification, evolutionary relationships, and significantly different species of microorganisms during the *suansun* fermentation process. It can be seen from Figure 9D that *Lactiplantibacillus, Lactococcus, Weissella, Limosilactobacillus, Leuconostoc, Paucilactobacillus, Furfurilactobacillus,* and other bacterial genera, although belonging to the larger group of lactic acid bacteria, have evolutionary differences which may lead to differences in their functions and metabolic pathways during the *suansun* fermentation process. *Lactobacillus, Weissella, and Lactococcus* were the main genera in *suansun* (Chen et al., 2023; Hu et al., 2023), which is similar to our research findings. *Lactobacillus plantarum* and *Limosilactobacillus reuteri* can increase the sourness of bamboo shoots and reduce bitterness respectively (Wang et al., 2023).

In order to further understand the influence of microbial communities in *suansun* on the production of key flavor compounds, a correlation network heatmap was drawn based on the Mantel test correlation test results, showing the correlation between 18 dominant bacterial genera and the content of 27 key flavor compounds (Figure 9E). The correlation analysis showed that a total of 23 key flavor compounds were highly significantly correlated with *Weissella* (17), *Limonolobacillus* (1), and *Actinmyces* (5), with *p* ≤ 0.001 (Table 3). *Weissella* and *Actinomyces*, which are both biomarkers of PWLS, significantly correlated with 22 key flavor compounds, accounting for 95.65%. For values *p* ≤ 0.01 to *p >* 0.001, there were five bacterial genera highly correlated with key flavor compounds, namely *Actinomyce*, *Weissella*, *Enterococcus*, *Limosilactobacillus,* and *Paucilactobacillus*. In addition to *Limonolobacillus*, *Paucilactobacillus* is a key biomarker for PW samples, while the remaining three belong to the PWLS treatment group. For values *p* < 0.05 to *p* > 0.01, there were five bacterial genera significantly associated with key flavor compounds, namely *Comamonas*, *Enterococcus*, *Weissella*, *Actinomyce*, and *Furfurilactobacillus*, all key microbial markers from PWLS. In addition, this study also found a positive correlation between the absolute dominant bacterial genus *Weissella* and *p*-cresol (*r* = 0.32, *p* = 0.018). Sun et al. also found that *Weissella and Lactococcus* were substantially linked to flavoring substances such hexanol, acetic acid, and *p*-cresol (Sun et al., 2023). *Weissella* is not only the dominant bacterium in the fermentation of *suansun*, but may also be the main genus of bacteria that produces key flavors in *suansun*. On the contrary, *Lactiplantibacillus*, which has the highest average relative abundance, has no statistically significant correlation with the 27 key flavor compounds, which may be one of the drawbacks of direct inoculation of a single strain. According to reports, the sensory quality of fermented foods, which are often treated with microorganisms, is altered by changes in the microenvironment (Liu et al., 2021). Additionally, scientists found that using several strains for synergistic fermentation results in a more robust flavor profile in addition to improving the fermentation process’ safety (Huang et al., 2021; Luo et al., 2020).

**Table 3:** 18 key flavor compounds significantly positively correlated with three bacterial genera.

No	RI	Compound name	Formula	CAS No	Genus	*r*	*p*
1	753	7-Hydroxy-7,8,9,10-tetramethyl-7,8-dihydrocyclohepta[d,e]naphthalene	C_18_H_20_O	1000110-34-9	*Weissella*	0.51	0.001
2	859	Oxalic acid, monoamide, n-propyl, propyl ester	C_8_H_15_NO_3_	1000309-64-1	*Weissella*	0.51	0.001
3	859	Oxalic acid, monoamide, n-propyl, hexyl ester	C_11_H_21_NO_3_	1000309-64-5	*Weissella*	0.54	0.001
4	859	2-Butanone, 3-methyl-	C_5_H_10_O	563-80-4	*Weissella*	0.54	0.001
5	2126	3-Methylpyridazine	C_5_H_6_N_2_	1632-76-4	*Weissella*	0.49	0.001
6	2126	Phosphonic acid, (p-hydroxyphenyl)-	C_6_H_7_O_4_P	33795-18-5	*Weissella*	0.57	0.001
7	1327	Propanoic acid, 2-oxo-, ethyl ester	C_5_H_8_O_3_	617-35-6	*Weissella*	0.64	0.001
8	1493	3,5-Dimethylpyrazole	C_5_H_8_N_2_	67-51-6	*Weissella*	0.63	0.001
9	1493	1H-Imidazole-4-ethanamine, beta.-methyl-	C_6_H_11_N_3_	24160-42-7	*Weissella*	0.61	0.001
10	1493	3,5-Dimethylpyrazole-1-methanol	C_6_H_10_N_2_O	85264-33-1	*Weissella*	0.58	0.001
11	786	Butane, 2-iodo-3-methyl-	C_5_H_11_I	18295-27-7	*Weissella*	0.39	0.001
12	999	Sulfurous acid, 2-ethylhexyl hexyl ester	C_14_H_30_O_3_S	1000309-20-2	*Weissella*	0.50	0.001
13	879	Hexane, 2-bromo-	C_6_H_13_Br	3377-86-4	*Weissella*	0.71	0.001
14	924	n-Hexane	C_6_H_14_	110-54-3	*Weissella*	0.40	0.001
15	1718	Tetradecane, 1-iodo-	C_14_H_29_I	19218-94-1	*Weissella*	0.39	0.001
26	1718	Nonane, 1-iodo-	C_9_H_19_I	4282-42-2	*Weissella*	0.44	0.001
17	1217	1-Pentanol	C_5_H_12_O	71-41-0	*Weissella*	0.35	0.001
18	1185	Pentanoic acid, 3-methyl-4-oxo-	C_6_H_10_O_3_	6628-79-1	*Limosilactobacillus*	0.70	0.001
19	1327	Propanoic acid, 2-oxo-, ethyl ester	C_5_H_8_O_3_	617-35-6	*Actinomyces*	0.62	0.001
20	1493	3,5-Dimethylpyrazole	C_5_H_8_N_2_	67-51-6	*Actinomyces*	0.63	0.001
21	1493	3,5-Dimethylpyrazole-1-methanol	C_6_H_10_N_2_O	85264-33-1	*Actinomyces*	0.60	0.001
22	879	Hexane, 2-bromo-	C_6_H_13_Br	3377-86-4	*Actinomyces*	0.67	0.001
23	1718	Nonane, 1-iodo-	C_9_H_19_I	4282-42-2	*Actinomyces*	0.78	0.001

In summary, PWLS treatment is the most optimal of the three fermentation methods. Direct fermentation of plant lactobacilli can play an important role in accelerating the maturation of *suansun*, which may be beneficial for commercial production. In subsequent direct fermentation studies, inoculation with composite lactic acid bacteria may be a better exploration model for achieving commercial production.

## 4 Conclusion

Using SBSE-GC-MS combined with electronic nose technology, a comparative analysis was conducted on 54 aroma components of *suansun* prepared by natural fermentation and direct fermentation. The results showed significant differences in the types and amounts of aroma components among the three types of *suansun*, and exhibited distinct fermentation mode characteristics. A total of 114 common aroma compounds were isolated and identified, including 31 esters, 16 alcohols, 16 hydrocarbons, 16 ketones, 9 acids, 7 aldehydes, 6 heterocyclic compounds, 4 phenols, 4 halogenated hydrocarbons, 3 amides, and 2 others. Esters, alcohols, and phenols contribute more to flavor formation in natural fermentation, while esters, aldehydes, ketones, and amides in direct-pitch fermentation. Based on the *p*-value and VIP value, 27 characteristic flavor compounds related to the aroma quality of *suansun* were further screened. Based on PCA analysis of 27 characteristic aroma compounds, the cumulative variance contribution rate of the first two PCs reached 90%. Based on the SVM model constructed by the first two PCs, a discriminant analysis was conducted on the fermentation method of *suansun*. The samples were randomly divided into a training set (70%) and a testing set (30%), with a discrimination rate of 100%. This indicates that the model is reliable and provides new ideas for the selection of fermentation methods for *suansun*.

The SBSE-GC-MS detection results showed that the main flavor compound categories increased nearly exponentially from natural fermentation day 14, reaching their peak at the fermentation 32 day, which is consistent with the changes in the electronic nose. The 27 key flavor compounds showed the most significant response under PWLS treatment, with *p*-cresol content increasing the most during the entire fermentation process, reaching over 600 times. The changes in colony structure during the fermentation process of bamboo shoots were analyzed using 16sRNA, and the results showed that the main dominant bacterial genus for the three fermentation methods were *Lactiplantibacillus, Lactococcus, Weissella,* and *Limosilactobacillus*. The abundance of the absolute dominant bacterial genus *Lactiplantibacillus* during direct fermentation rapidly increased on the 2nd day, reached a peak on fermentation day 14, significantly higher than with natural fermentation, then gradually decreased, reaching its lowest point on fermentation day 32. This indicates that direct fermentation of plant lactobacilli can play an important role in accelerating the maturation of bamboo shoots. However, among the 23 matched pairs where the dominant bacterial genera of the three fermentation methods were significantly positively correlated with key flavor compounds, PWLS accounted for 95.65%, of which 17 key characteristic flavor compounds were significantly positively correlated with *Weissella*. *Weissella* is not only the dominant bacterium in the fermentation of bamboo shoots, but also the main genus of bacteria that produces key flavors in bamboo shoots. There is no statistically significant correlation between *Lactiplantibacillus* and the 27 key flavor compounds. Therefore, using direct injection and inoculating with composite lactic acid bacteria may be an optimal model for achieving commercial production.

## Data Availability

The original contributions presented in the study are included in the article/supplementary material, further inquiries can be directed to the corresponding author.
